# A Spitzoid Tumor dataset with clinical metadata and Whole Slide Images for Deep Learning models

**DOI:** 10.1038/s41597-023-02585-2

**Published:** 2023-10-16

**Authors:** Andrés Mosquera-Zamudio, Laëtitia Launet, Rocío del Amor, Anaïs Moscardó, Adrián Colomer, Valery Naranjo, Carlos Monteagudo

**Affiliations:** 1 Pathology Department Hospital Clínico Universitario de Valencia, Universidad de Valencia, Valencia, Spain; 2 INCLIVA, Instituto de Investigación Sanitaria, Valencia, Spain; 3 Instituto Universitario de Investigación en Tecnología Centrada en el Ser Humano, HUMAN-tech Universitat Politècnica de València, Valencia, Spain; 4 valgrAI: Valencian Graduate School and Research Network of Artificial Intelligence, Valencia, Spain

**Keywords:** Cancer imaging, Cancer imaging

## Abstract

Spitzoid tumors (ST) are a group of melanocytic tumors of high diagnostic complexity. Since 1948, when Sophie Spitz first described them, the diagnostic uncertainty remains until now, especially in the intermediate category known as Spitz tumor of unknown malignant potential (STUMP) or atypical Spitz tumor. Studies developing deep learning (DL) models to diagnose melanocytic tumors using whole slide imaging (WSI) are scarce, and few used ST for analysis, excluding STUMP. To address this gap, we introduce SOPHIE: the first ST dataset with WSIs, including labels as benign, malignant, and atypical tumors, along with the clinical information of each patient. Additionally, we explain two DL models implemented as validation examples using this database.

## Background & Summary

*Sophie Spitz* was an American pathologist recognized for describing in 1948 a specific type of melanocytic tumor in 13 children and young patients, which she named “juvenile melanomas”^1^. According to her description, this type of neoplasm has the peculiarity of resembling melanoma due to its histological features, but the clinical outcome is usually benign. Out of the 13 cases that she studied, only one died due to malignant clinical behavior, while the remaining cases had favorable outcomes. This study has been cited more than 800 times and has challenged how melanomas and nevi have been diagnosed since then. Therefore, as a tribute to her work, melanocytic tumors with these histopathologic features are nowadays known as *Spitzoid tumors* (ST).

**Fig. 1:**
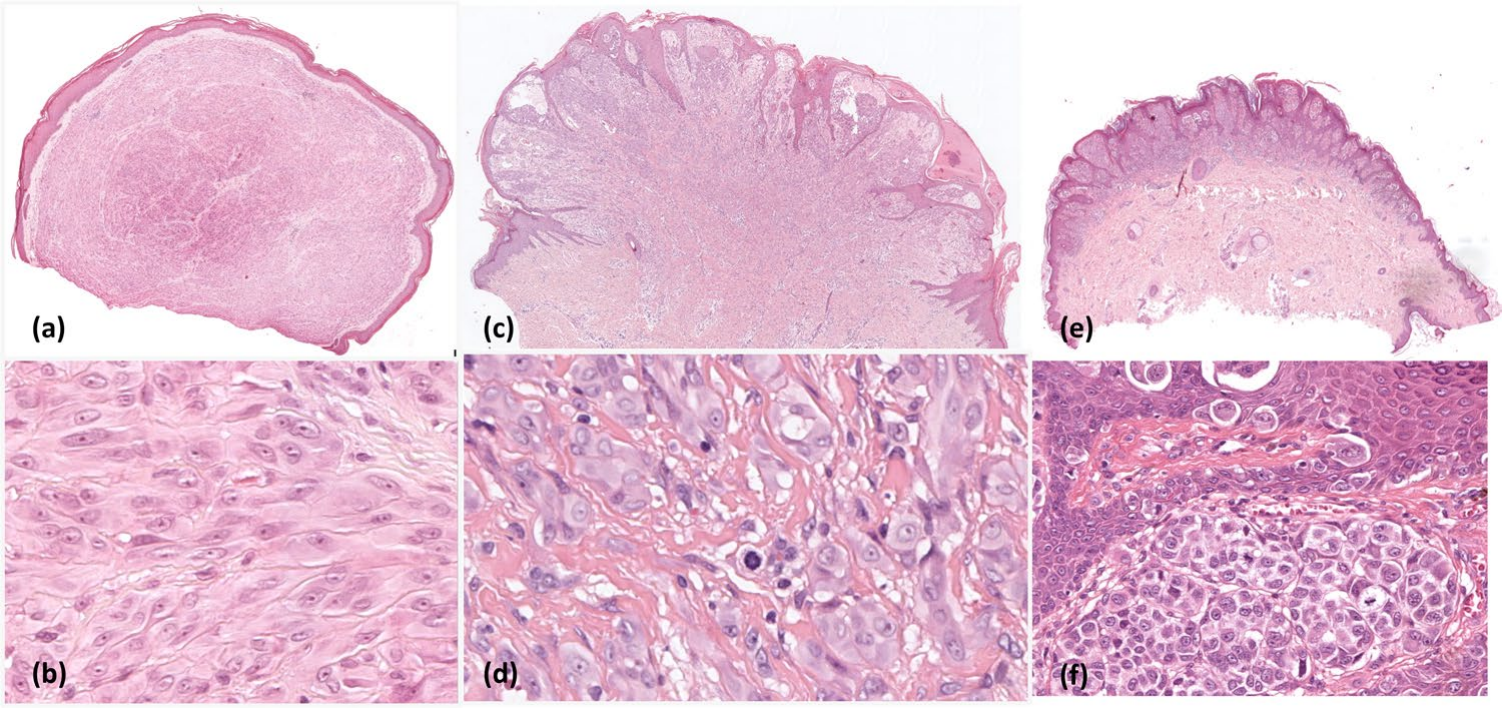
Representative patches extracted from WSIs presenting ST; (**a**-**b**): SN containing large uniform melanocytic cells devoid of mitotic activity in an organized fashion; (**c**-**d**) STUMP with deep mitosis; (**e**-**f**): SM with numerous mitotic figures and pagetoid spread.

The STs, which represent 1-2% of all melanocytic tumors, are one of the most challenging entities in histopathological diagnosis^2,3^. The discrepancy between the histopathological appearance and the clinical evolution can lead to misdiagnosis and result in under or overtreatment^4^. STs are defined as melanocytic proliferations with large epithelioid or spindle-shaped melanocytes with large nuclei, vesicular chromatin, and prominent nucleoli^2^. This entity is divided into three categories. The benign group is called *Spitz Nevus* (SN), the malignant type *Spitzoid Melanoma* (SM), and the third one is an intermediate category due to its prognostic uncertainty, called *Spitzoid Tumor of Unknown Malignant Potential* (STUMP) or *Atypical Spitz Tumor*^2^. (See Fig. 1 to see representative examples of ST).

Researchers have spent decades attempting to clarify the differences between these three categories and trying to improve prediction accuracy^5^. Nowadays, the pathologist’s interpretation of Hematoxylin and Eosin (H&E) stained glasses remains the gold standard in diagnosis^2,6^. Despite the high interobserver variability in diagnosing ST, certain histological characteristics support its categorization^7^. Table 1 summarizes the main features distinguishing the three types of STs.

**Table 1:** Main histological features in the diagnosis of Spitz Tumors.

Feature	Spitz Nevus	STUMP	Spitz Melanoma
**Dimensions**	<5 to 6 mm	5 - 10 mm	>5 mm often >10 mm
**Symmetry**	Yes	Rare	No
**Circumscription**	Well demarcated	Well or poorly demarcated	Poorly demarcated
**Ulceration**	No	Very Rare	May be present
**Mitotic rate**	<2 / mm^2^	2-6 / mm^2^	>6 mm^2^
**Atypical Mitosis**	No	No	Yes
**Deep Mitosis**	No	May be present	Yes
**Necrosis**	No	No	Yes
**High-grade Nuclear Atypia**	No	No	Yes
**Kamino Bodies**	Yes	Rare	Extremely Rare
**Pagetoid Spread**	If any, central and Focal	Greater than SN	Extensive

Many molecular studies trying to ensure the nature of an ST and approximate its clinical behavior have been performed. The most frequent alterations (50%) in spitzoid melanocytic tumors are genetic rearrangements derived from kinase fusions of ALK, ROS1, BRAF, RET, MET, NTRK1, NTRK3, MAP3K8, or MAP3K3^8,9^. HRAS mutations can also be found in these tumors. Furthermore, the genetic studies have helped to separate different entities, like BAP1-inactivated melanocytic tumors, which were initially considered a subgroup of ST since their histologic features overlap with STUMP^10–12^. Also, the presence of TERT promotor mutations favors the malignant potential of these neoplasms^2,8,13^. Notwithstanding all these findings, there is still no genetic trait that clearly differentiates these tumors according to their benign or malignant clinical behavior, especially in STUMPs, where their correct diagnosis remains a conundrum, and the interpretive diagnosis by the pathologist in the H&E slide prevails over other complementary diagnostic techniques.

In the Digital Pathology era, novel and promising advances have been made by implementing deep learning (DL) models for image recognition in histological whole slide images (WSIs)^5,14,15^. A WSI is a digital scanning technology that captures and converts glass slides for use in pathology, histology, and other healthcare fields into high-resolution digital images. A digital image can be viewed, analyzed, and shared electronically, which makes diagnosis, research, and collaboration between healthcare professionals more efficient and accurate. It is estimated that by 2030, several algorithms will be part of the pathology laboratory workflow, where the diagnostic accuracy will increase, and the diagnosis and tumor grading will be more standardized by decreasing subjectivity, especially in tumors of high diagnostic complexity that lead to crucial inter-observer variability among other improvements in the pathology field^5^.

Most studies on DL models for melanocytic tumors have utilized clinical and dermatoscopic images^16–19^. However, a small group of studies has focused on using WSIs and comparing the performance of pathologists vs. DL models regarding diagnostic accuracy, prognosis prediction, and histological feature detection^19^. Only three studies have focused on Spitz tumors but excluded STUMP^20–22^. Hart *et al*.^22^ developed a convolutional neural network to differentiate between ST and conventional melanocytic lesions with a classification accuracy at the patch level of 0.99 +0.2. The other two were done by Del Amor *et al*. The first one used a semi-weakly supervised DL framework based on inductive transfer learning to differentiate malignant and benign samples in Spitzoid lesions^20^ achieving an accuracy of 0.92 and 0.80 for the source and the target models, respectively. The other two studies presented a multi-resolution framework to automatically assess morphological features at different resolution levels and combine them to provide a more accurate diagnosis, demonstrating that this method outperforms single-resolution frameworks in ST classification^21^.

To the authors’ best knowledge, there is no publicly available dataset that allows the study of ST in WSIs. In this paper, we introduce the dataset previously used by Del Amor *et al*. in their publications regarding ST^20,21^. This dataset of ST cases (called “SOPHIE” in remembrance of Sophie Spitz) will include Hematoxylin and Eosin (H&E) stained WSI and clinical information, which will help researchers to explore and develop methods to improve diagnosis and classification of ST. This dataset comprises 61 H&E stained ST WSIs from 58 patients with clinical information. Additionally, the dataset includes a Python script that was developed for the semi-weakly supervised DL study done by Del Amor *et al*.^20^.

## Methods

### Study approval

The Ethics Committee of the University Clinic Hospital of Valencia approved the study (n^°^ 2020/114) as part of the Clarify Project (from the European Union’s Horizon 2020 Program for Research and Innovation, under the Marie Skłodowska Curie grant agreement No. 860627), which was conducted in conformity with the principles of the Declaration of Helsinki. The dataset of this retrospective study was conducted following the ethical guidelines and regulations set forth by our Institutional Review Board (IRB) which required the patient informed consent including data sharing and open access publication. We ensured that all data used in this research was de-identified and handled in a secure and confidential manner to protect the participants’ rights and welfare.

### Selection and preparation of the slides

The SOPHIE dataset was collected at the *Department of Anatomical Pathology* of the *Hospital Clínico Universitario de Valencia - HCUV* (Valencia, Spain) between the years 1988 and 2020. A total of 61 H&E slides from 58 patients were collected from the hospital archives according to the pathology reports.

#### Expert labels

The slides were re-evaluated by a dermatopathologist (CM) with more than 30 years of experience in the field and by two general pathologists (AMZ, AM) in order to confirm the diagnosis of every case and to label each image. Specifically, 30 of the 58 patients under study were diagnosed as SN, 18 as SM, and 10 as STUMP.

#### Digitization and Pre-processing

The formalin-fixed paraffin-embedded (FFPE) tissue blocks and slides of all selected cases were collected from the institution’s archives.

After selecting the slides for digitization, a qualified pathologist (AMZ, AM) oversaw the process. Once the digitization was completed, the same pathologists thoroughly assessed each image to ensure it met the necessary quality standards. If a WSI did not meet the pathologists’ criteria, they took corrective actions. This involved either re-scanning the original slide or, if necessary, using the corresponding FFPE block to obtain a new slide for digitization..

The digitization process was carried out using Roche’s scanner, Ventana iScan HT, equipped with a 40 × objective lens (0.227 M/pixel) and saved in.tif file format. The digitization covered a maximum magnification of 40 × , including all levels down to 5 × .

### Clinical Data Acquisition

The clinical data were obtained from the records in the HCUV’s hospital information system, with the previously signed consent of each patient. The variables included in the database are shown in Table 2. Personal identifiers were removed, and data aggregation techniques were employed to prevent the identification of individual patients.

**Table 2:** Clinical Variables.

Field	Explanation	Value
Age (years)	Age at the time of diagnosis	Years
Sex	Type of sex	1. Female
		2. Male
Tumor location	Location of the primary tumor	1. Head and neck
		2. Trunk
		3. Upper limb and shoulder
		4. Lower limb and hip
		5. Not recorded or specified
Local recurrence	New growth at the primary site after excision	1. Absent
		2. Present
Regional relapse	Spread into near lymph nodes or tissues	1. Absent
		2. Satellite/in transit metastasis
		3. Lymph node metastasis
		4. Lymph node AND satellite/in transit metastasis
Distant metastasis	Tumor dissemination to distant sites	1. Absent
		2. Skin, soft tissue and/or non-regional lymph node
		3. Lung
		4. Non-Central Nervous System and visceral sites
		5. Central Nervous System
Follow up	Follow up according to the last date	1: Alive and well
		2: Alive with local recurrence
		3: Alive with regional relapse
		4. Alive with distant metastasis
		5: Dead of tumor
		6: No follow up

## Data Records

The complete SOPHIE dataset is available at the public figshare repository^23^. The dataset consists of three components. There is a file containing the WSIs, a spreadsheet referred to as “SOPHIE_DATASET.xlsx” which includes the clinical data tabulated according to Table 2 along with the histopathological diagnosis for each case, and a file with the codes associated with the dataset written in Python referred to as “paper_dataset.zip”.

### Image Data

WSIs are grouped into three categories according to the histopathological diagnosis, and each file is named according to the following format: Spitz Nevus (SN_00XX), Spitzoid Melanoma (SM_00XX), and Spitzoid Tumor of Unknown Malignant Potential (STUMP_00XX).

In the SM group, there are two notable cases that involve multiple Whole-Slide Images (WSI).

SM_0015: This case is represented by two WSIs, “SM_0015A” and “SM_0015B.” Each image corresponds to different regions within the same tumor because the size of the tumor overpasses the dimensions of one FFPE block. By having multiple WSIs, we can gain insights into the spatial variation and heterogeneity present within this specific tumor, enhancing our understanding of its characteristics.

SM_0016: In this case, we have “SM_0016A” representing the primary tumor. Additionally, “SM_0016B” and “SM_0016C” showcase the lymph node metastases from the same patient. This multi-slide representation allows us to explore the metastatic spread and identify potential differences between the primary tumor and the metastatic sites.

By including multiple WSIs for these cases, we aim to provide a more comprehensive view of tumor behavior. This explicability allows researchers and clinicians to analyze and interpret the dataset more effectively, contributing to a deeper understanding of the complexities in the classification and behavior of SM cases.

## Technical Validation

**Fig. 2:**
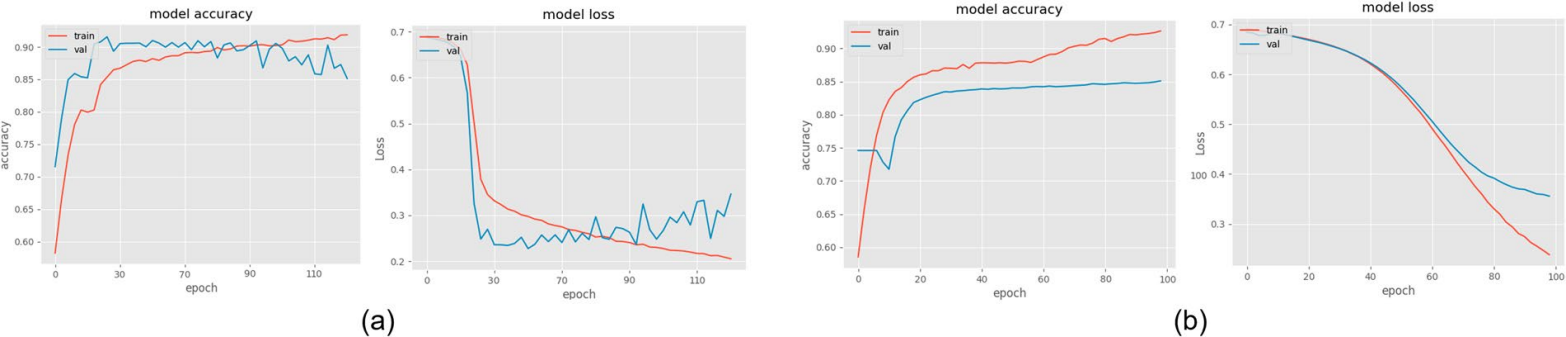
Learning curves for the source and target models. (**a**) Accuracy and loss for the source model trained at the patch level. (**b**) Accuracy and loss for the target model trained at the biopsy level.

To validate the dataset proposed in this paper, we present a CNN-based approach for Spitzoid lesions analysis that leverages the SN and SM WSIs at hand along with the experts’ annotations, previously published^20^. The experiments were two-fold, starting with a source model to identify tumor regions trained with experts’ segmentation, and a target model for the overall classification of WSIs into benign or malignant, pre-trained with the weights of the source model to provide it with prior histological knowledge. Note that the presented models were trained following a 4-fold cross-validation strategy to optimize both models and were tested on 30% of the overall dataset, that is to say, 15 images. The learning curves for the source and the target models are shown in Fig. 2.

### Data pre-processing

**Fig. 3:**
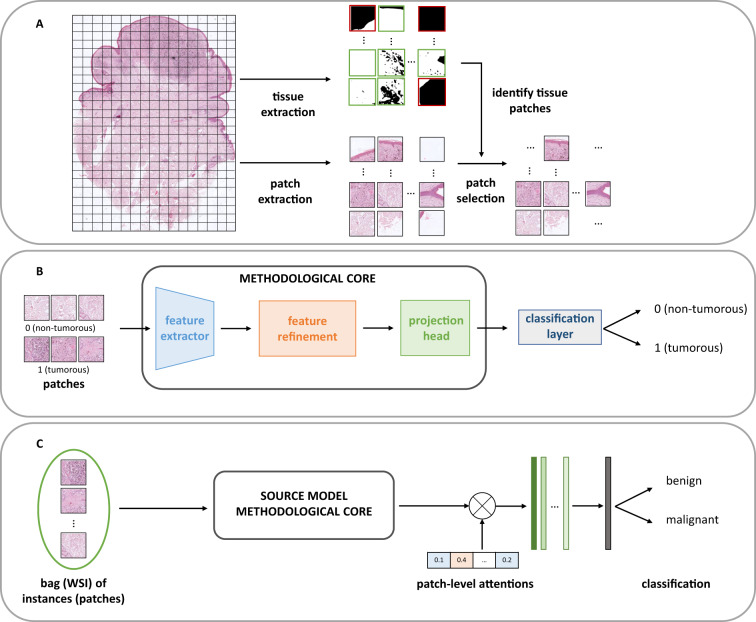
Technical validation experiments. (**A**) Data pre-processing carried out before training models. (**B**) Source model: ROI identification at patch-level; (**C**) Target model: slide-level classification using bags of instances, leveraging the pre-trained source model. Note that the methodological core is similar in both approaches.

In this approach, the WSIs were accessed at a 10 × resolution. Because of the particularly large size of WSIs, these were first cropped into smaller patches of 512 × 512 pixels, each with a 50% overlap. The Otsu’s thresholding method was then applied to the magenta channel of the images to separate tissue from the background, allowing to discard patches with less than 20% of tissue and thus reducing possible noise in the input data. This process is depicted in Fig. 3-(A).

### Patch-level ROI identification

For this first validation task to identify patches with tumor to select regions of interest (ROIs) using the pixel-level annotations, we refined a CNN feature extractor based on the VGG16 architecture pre-trained in ImageNet^24^. In particular, the first convolutional block of the architecture was frozen, while the next blocks were re-trained to fit the specific application, and an attention module as proposed in^20^ was added to the output feature map to focus on the key features^25^. The tumorous patches identification is then determined with the projection head module, consisting of a global max pooling layer.

**Fig. 4:**
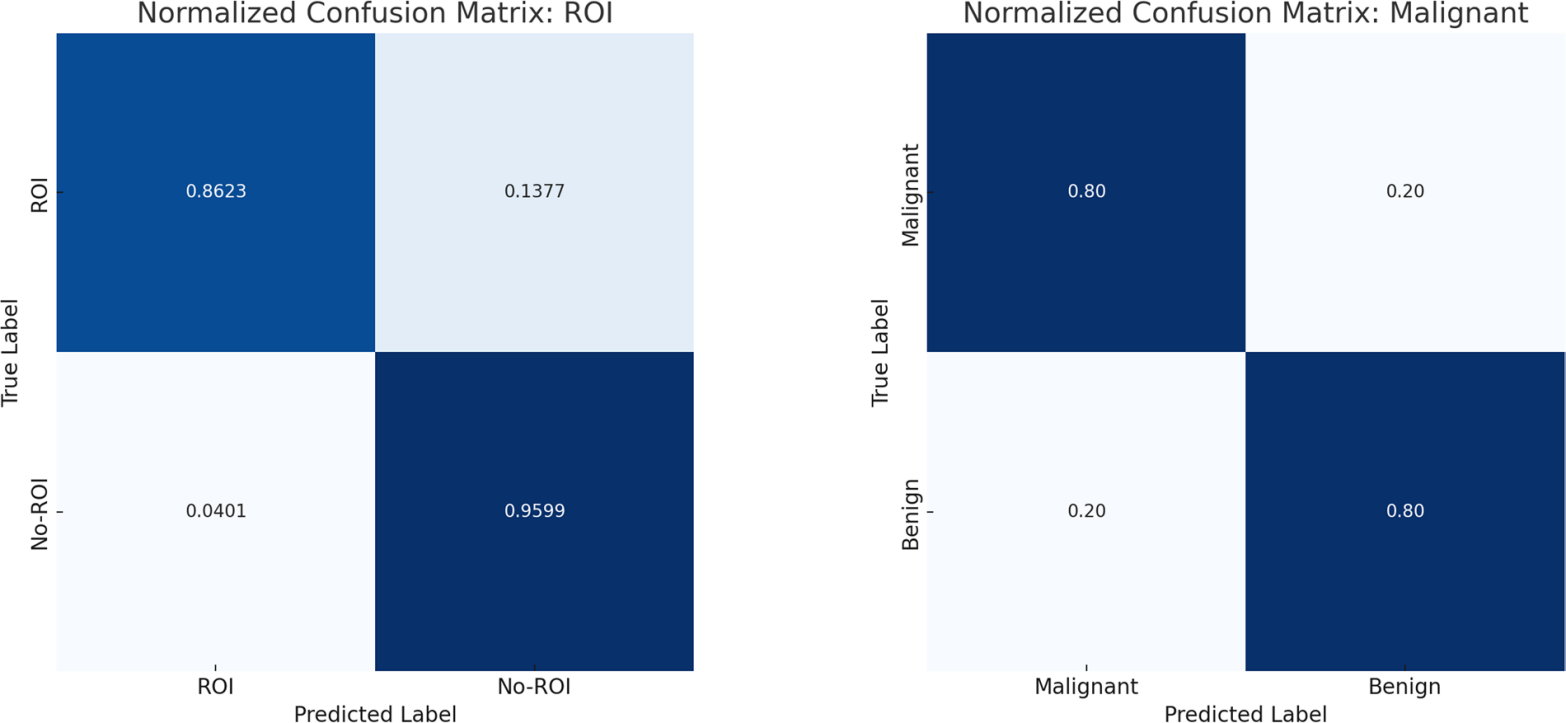
Normalized confusion matrix for the source and target models. (**a**) Confusion matrix for the source model trained at the patch level. (**b**) Confusion matrix for the target model trained at the biopsy level.

The ROI identification source model was trained for m epochs with a batch size of 64, using the stochastic gradient descent optimizer with a learning rate of 0.001 to minimize the binary cross-entropy loss function. In this approach, we reached an accuracy of 0.9231 on the test set, and 0.9285, 0.9202, 0.8942 for the sensitivity of the malignant class, specificity and F1-score metrics, respectively. The confusion matrix of the ROI model is shown in Fig. 4(a).

### WSI-level tumor classification

The second validation task consisted of performing a WSI-level classification of the Spitzoid lesions into benign or malignant. For this purpose, we trained a classification model under a multiple instance learning (MIL) scenario where each WSI corresponds to a bag composed of *n* instances, i.e., the tumor patches within the WSI, predicted with the previously described patch-level ROI prediction model.

As shown in Fig. 3-(B), to train this target model, we used the previous source model weights to leverage prior knowledge of histology-specific features and transfer it to the new model application with inductive learning, then fine-tuning it for this specific application. Thanks to this backbone, an embedding vector was generated for each instance in a bag and combined with the tile-level attention to weigh the patches according to their importance in the final WSI-level prediction. These patch embeddings were then aggregated with an attention-based trainable aggregation function^26^ to classify the entire WSI into benign or malignant.

This WSI-level classification model was trained for 100 epochs with the same learning rate and loss function as the ROI identification model presented in the previous section, with a batch size of 1, that is to say, one slide per batch. This approach achieved an accuracy of 0.80 on the test set for WSI-level classification, with a sensitivity of malignant cases, specificity and F1-score of 0.67, 0.89, and 0.73, The confusion matrix of the WSI model is shown in Fig. 4(b).respectively.

## Usage Notes

The choice of using 10x resolution over 20x in our model is based on computational efficiency and clinical relevance. While 20x offers mode detail, 10x provides a broader field-of-view, allowing for comprehensive tissue analysis. Furthermore, 10x simulates the pathologist’s approach for the initial screening of the slide. Notably, even at 10x, our model effectively identifies tumors, regardless of their size. This approach ensures a balance between efficiency, clinical orientation, and diagnostic accuracy.

### Limitations

The dataset has some limitations. Firstly, the number of images is relatively small compared to other types of tumors, considering that the prevalence of these tumors is only 1% of all melanocytic tumors^2^. Moreover, the percentage of images representing STUMP or SM is even lower.

While the technical validation of this dataset focuses solely on SN and SM WSIs, it successfully demonstrates the effectiveness of DL models in this limited context. By showcasing the utility of DL models with this subset, it highlights the potential for applying similar approaches to include STUMP data and other related cases. As such, this validation lays the groundwork for future investigations that can encompass a broader range of melanocytic tumors, further expanding the dataset’s applicability and potential impact in the field.

Additionally, it is important to acknowledge that these images were digitized using a single scanner, which could potentially impact the generalizability of the results.

Despite these limitations, this dataset still provides valuable insights into the rare category of melanocytic tumors and can serve as a foundation for further research and analysis.

## Data Availability

The file code is in the figshare repository^23^ as “paper_dataset.zip”. They are mainly two folders. The folder “First_step” has the file “Train_Patch_level_model.py”. This file contains the necessary statements to perform patch-level classification for regions of interest and non-interest. In the folder “Second_step,” three files are located. The file “WSI_prediction_MIL” contains the main functionalities for training an algorithm based on Multiple Instance Learning (MIL). This script calls several functions stored in the “DataGenerator” and “utils_1.” files. The file “DataGenerator” holds the necessary functions for loading data in the form of bags required for a Multiple Instance Learning algorithm. In the “utils_1” folder, all the necessary functions for extracting metrics, calculating loss, and more can be accessed. These functions are utilized in the “WSI_prediction_MIL” file.
